# Spatio-Temporal Dynamics of Entropy in EEGS during Music Stimulation of Alzheimer’s Disease Patients with Different Degrees of Dementia

**DOI:** 10.3390/e24081137

**Published:** 2022-08-17

**Authors:** Tingting Wu, Fangfang Sun, Yiwei Guo, Mingwei Zhai, Shanen Yu, Jiantao Chu, Chenhao Yu, Yong Yang

**Affiliations:** School of Automation, Hangzhou Dianzi University, Hangzhou 310018, China

**Keywords:** EEG, Alzheimer’s disease, different degree of dementia, music stimulation, permutation entropy

## Abstract

Music has become a common adjunctive treatment for Alzheimer’s disease (AD) in recent years. Because Alzheimer’s disease can be classified into different degrees of dementia according to its severity (mild, moderate, severe), this study is to investigate whether there are differences in brain response to music stimulation in AD patients with different degrees of dementia. Seventeen patients with mild-to-moderate dementia, sixteen patients with severe dementia, and sixteen healthy elderly participants were selected as experimental subjects. The nonlinear characteristics of electroencephalogram (EEG) signals were extracted from 64-channel EEG signals acquired before, during, and after music stimulation. The results showed the following. (1) At the temporal level, both at the whole brain area and sub-brain area levels, the EEG responses of the mild-to-moderate patients showed statistical differences from those of the severe patients (*p* < 0.05). The nonlinear characteristics during music stimulus, including permutation entropy (PmEn), sample entropy (SampEn), and Lempel–Ziv complexity (LZC), were significantly higher in both mild-to-moderate patients and healthy controls compared to pre-stimulation, while it was significantly lower in severe patients. (2) At the spatial level, the EEG responses of the mild-to-moderate patients and the severe patients showed statistical differences (*p* < 0.05), showing that as the degree of dementia progressed, fewer pairs of EEG characteristic showed significant differences among brain regions under music stimulation. In this paper, we found that AD patients with different degrees of dementia had different EEG responses to music stimulation. Our study provides a possible explanation for this discrepancy in terms of the pathological progression of AD and music cognitive hierarchy theory. Our study has adjunctive implications for clinical music therapy in AD., potentially allowing for more targeted treatment. Meanwhile, the variations in the brains of Alzheimer’s patients in response to music stimulation might be a model for investigating the neural mechanism of music perception.

## 1. Introduction

Alzheimer’s disease (AD) is a chronic disease caused by the decline of the central nervous system. It is characterized by cognitive impairment, memory loss, and even psychological disorders. Due to the aging of the world population, the number of people suffering from Alzheimer’s disease is increasing year by year throughout society [1,2]; 2% of the elderly population over 60 years of age in Western countries have a probability of developing Alzheimer’s disease, and the number of elderly people suffering from Alzheimer’s disease in China has exceeded 5 million, which is roughly equivalent to 25% of the global number of AD patients. As the pathological mechanism of Alzheimer’s disease remains unclear, there is little medical treatment that can completely cure the disease. Current treatments focus on symptom relief to prevent further progression of the disease, for example, pharmacological treatments to control the disease [3]; however, the safety of pharmacological treatments remains to be determined.

Among non-pharmacological treatments, music is a common complementary treatment for Alzheimer’s disease [3]. Music is a common language that is easily understood by all human beings and which can be understood by people with different levels of education and life backgrounds. Music can change the physical and mental state of patients [4], helping them to achieve physical and mental pleasure and relaxation, helping to relieve the stress caused by adverse psychological factors, and ensuring the safety of treatment [5]. Numerous studies have shown that music has cognitive as well as emotional benefits for Alzheimer’s patients [6,7,8,9,10,11].

Because Alzheimer’s disease can be classified into different levels of dementia (mild, moderate, and severe) according to its severity [12], a great deal of research involving music treatment for AD patients has adopted behavioral scales for analysis, including the Mini-Mental State Examination (MMSE), Behavioral and Psychological Symptoms of Dementia (BPSD), and others. The MMSE can systematically and accurately reflect the degree of cognitive decline as well as the level of intelligence, and has been revised by Ming-Yuan Chang. The scale has profound guiding significance for neuropsychological research and clinical psychological diagnosis. It has been widely promoted and used because of its simplicity and ease of implementation, and has become the preferred scale for diagnosing the degree of dementia. The MMSE score is highly correlated with educational level, with one point for a correct answer and zero points for a wrong or unknown answer. The normal threshold classification criteria are: illiterate > 17; primary > 20; and secondary and above > 24. The BPSD is an assessment of the mental health status of Alzheimer’s patients, both to determine the presence of the disease and to detect its frequency and severity. These assessments can evaluate the effectiveness of treatment over a period of time.

Behavioral scales are widely utilized in the assessment of the cognitive and emotional status of Alzheimer’s patients thanks to their numerous advantages. Sylvain Clément et al. adopted BEHAVE-AD along with MMSE assessment in a study of music intervention in patients with severe Alzheimer’s disease [8], finding that severe patients had reduced anxiety during music intervention. Johnson JK et al. found in their study that AD twins who listened to Mozart’s piano concerto showed a significant improvement in spatio-temporal awareness [10]. Li CH et al. used external headphones for mild AD patients, finding that six months of music intervention resulted in less decline in scores on various cognitive-related behavioral scales than the no-music control group. Cognitive performance was better in the music therapy group compared to the no-music control group [11]. All of the above research illustrates the effectiveness of music therapy for mild-to-moderate or severe patients using behavioral scale scores.

However, behavioral scale scores are influenced by subjective factors, while the deeper mechanisms of brain responses during musical stimulation require objective quantitative indicators to be investigated. Therefore, it is necessary to utilize quantitative methods to assess the level of cognition in Alzheimer’s disease. EEG, as a traditional method for clinical examinations and experiments, is an objective and quantitative method of analysis. EEG processing currently includes two main methods, linear and nonlinear analysis [13]. The brain power spectrum reflects the energy information of each frequency band of EEG and is a linear way of a representing human brain activity. Power spectrum analysis is now widely used as well to study changing patterns in the brain; for example, it can be used to characterize brain changes in epileptic patients before and after treatment, as well as changes in brain wave frequencies in subjects during different sleep stages [14]. In addition, the brain is usually seen as a complex nonlinear system due to the non-stationarity of its activity [15]. The nonlinear dynamical model is the most widely used approach in current studies of neuro-electrical signals. As early as the 1990s, nonlinear dynamics research has been conducted in the field of neuro-electrophysiology. According to Professor Theiler, nonlinear dynamics features can represent the intensity of neuronal activity in brain waves and brain regions, allowing the brain to be viewed as a very complex chaotic system [16]. Kang states that while most of the traditional methods of EEG interpretation involve time series analysis and spectral analysis [14], current nonlinear analysis methods have been shown to possess great superiority in terms of increased detection rates. According to Stam [15], methods involving nonlinear time series have been applied to the detection of neuro-electrical and muscle electrical signals; for example, researchers have tested EEG and EMG signals in healthy subjects before stimulation and without a task, then during a period of cognitive processes with tasks to complete in order to aid in detection of pathological states. It is evident that EEG analysis methods have been widely used in established studies. EEG analysis has previously been involved in studies of music therapy for AD patients with different degrees of dementia. For example, in Alexie’s study, based on EEG recordings it was found that all AD patients had improved emotion and memory with an approach combining music and virtual reality technology [9].

In the relevant studies mentioned above, regardless of whether the method of analysis involved behavioral scales or EEG analysis, few studies have systematically compared differences in brain responses in AD patients during music therapy by the patients’ different degrees of dementia. Current research has rarely investigated whether there are differences in brain response to musical stimulation in AD patients with different degrees of dementia, which could provide a theoretical basis for music therapy in Alzheimer’s disease. Therefore, it is necessary to compare the differences in brain response during musical stimulation in Alzheimer’s disease patients with different degrees of dementia. Our study has guiding implications for the use of clinical music therapy for Alzheimer’s disease.

## 2. Methods

### 2.1. Patient Preparation

A total of 49 subjects were examined in this study, including 17 in the mild-to-moderate groups, 16 in the severe group, and 16 in control group.

Patients were recruited from the Xiangshu Geriatric Hospital in Zhejiang Province, China. Cases assessed by experienced neurologists were based on the Criteria for the clinical diagnosis of Alzheimer’s Disease (AD), which were established by the National Institute of Neurological and Communicative Disorders and Stroke (NINCDS) and the Alzheimer’s Disease and Related Disorders Association (ADRDA) workgroup in 1984 [17]. The Mini-Mental State Examination (MMSE) and Behavior and Psychological Symptoms of Dementia (BPSD) scores were adopted. Professional scorers from Xiangshu Geriatric Hospital in Zhejiang Province conducted the MMSE and BPSD measurements prior to the music stimulation experiment. The inclusion criteria for the mild-to-moderate group were MMSE ≥ 13 and meeting the criteria for NINCDS-ADRDA. The inclusion criteria for the severe group were MMSE < 13 and meeting the criteria for NINCDS-ADRDA. The exclusion criteria included: (1) a history of other brain diseases, such epilepsy and vascular dementia; (2) a history of other mental illness, not including behavioral and psychological symptoms of dementia; (3) abnormal hearing; (4) bedridden patients. The regional review board approved the use of human participants in this study. Family members and patients signed written informed consent forms before participation. The study was approved by the ethics committee of Xiangshu Geriatric Hospital, Hangzhou, China in accordance with the Helsinki Declaration. The patient information is summarized in Table 1.

**Figure 1:**
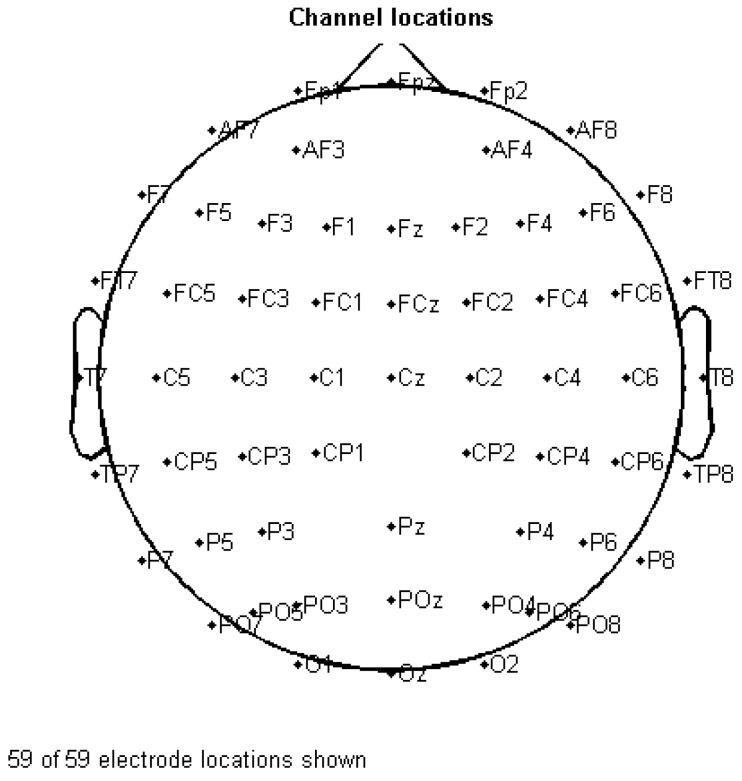
Electrode placement of Neuracle (W64; China) based on the 10–20 general international standard lead system. (Note: F: frontal; C: central; O: occipital; P: parietal; T: temporal; FP: frontal-parietal; AF: pre-frontal; FC: frontal-central; FT: frontal-temporal; CP: central-parietal; TP: temporal-parietal; PO: parietal-occipital. Odd numbers such as 1, 3, 5, 7 represent left; even numbers such as 2, 4, 6, 8 represent right; “z” represents central axis.).

EEG signals were recorded in single electrode channel mode using the Neuracle EEG system (W64; Neusen, Pinghu City, China). Electrodes were placed over the entire head according to the 10–20 general international standard lead system, which is shown in Figure 1. A total of 59 EEG channels recorded the signals, as another five channels of this EEG system involved the ocular electrical signal and electromyography (EMG) signal.

Signals were digitized at a sampling of 1000 Hz, and the electrode impedance was <500 Ω. For music stimulation, the Chinese classical music piece “Jasmine” was truncated into voice fragments and played for 60 s as the music stimulation. This piece was chosen because “Jasmine” is a household and familiar piece of music, and it was learned through clinical interviews that “Jasmine Flower” was familiar to all subjects in our study.

### 2.2. EEG Signal Preprocessing

#### 2.2.1. Filtering the Data

The processing environment was MATLAB R2016b with the EEGLAB tool kits (University of California San Diego, San Diego, CA, USA). The power frequency was eliminated using a 50 Hz notch filter, and data were filtered from 0.5–80 Hz using a band pass filter [18].

#### 2.2.2. Independent Component Analysis

The eye activity of AD patients is uncontrollable, which causes the existence of electro-oculogram (EOG) artifacts in EEG signals. Independent Component Analysis (ICA) was therefore used to remove EOG artifacts from the EEG signals. The basic model of ICA was as follows [19]:

(1) $$z\left(t\right)=A_{\delta }\left(t\right)+v\left(t\right)$$

(2) $$x\left(t\right)=A_{\delta }\left(t\right)$$

where *Z*(*t*) is the signal including the noise, *v*(*t*) is the noise, 

and $x(t)={\left[x_{1}(t),\cdots x_{m}(t)\right]}^{T}$, $m\in N^{*}$ is the signal without the noise. Formula (2) can then be substituted into Formula (1) to obtain Formula (3):

(3) z(t)=x(t)+v(t)

(4) zi(t)=xi(t)+vi(t)

where for the above Formulas (3) and (4) *i* = 1, …, *m*.

To date, the ICA algorithm has been widely used in various studies. Many investigators have used ICA to remove muscle activity noise from EEG signals, and have found that the ocular electrical signal and EMG signal can be separated as well [20,21].

The ICA steps used to remove noise and EOG artifacts were as follows. First, the filtered EEG signals were centralized so that every signal source had a mean value of 0. To make the signal variance 1 and to eliminate the correlation of signals, Principal Component Analysis was used to perform the whitening processing. Second, a distributed parallel mode was used to extract each independent component from the mixed signals so that every component was separated. Third, after separating each signal source, a topographical map of the brain was analyzed. Signal sources that have been shown to be active in eyes and ears in the topographical map were considered to be electro-ocular and electromyography signals which should be rejected. After removing the noise, the remaining components were reconstructed to obtain clear EEG signals without noise [21,22].

#### 2.2.3. Nonlinear Dynamics: PmEn, SampEn, LZC

Nonlinear dynamic analysis involves many indicators, including the entropy and complexity [23] as well as the correlation dimension and Lyapunov exponents [24,25]. Considering that the correlation dimension and Lyapunov exponents require large datasets and strict dimensions [26], entropy and complexity are suitable options because of small dataset demand and high computation speed. Among entropy features, PmEn and SampEn were used in this study. PmEn measures the complexity of the signal, with the advantage of being simple, fast, and having a strong anti-noise property [26]. PmEn was therefore used to analyze dynamic changes during music stimulation for AD patients with different degrees of dementia.

Permutation entropy is a method of measuring the complexity of a time series [27]. First proposed by Lempel, it has high robustness and is widely used in sequence complexity and nonlinear analyses. The one-dimensional sequence of the original signal was set to include *N* points, defined as x(1),x(2), ⋯⋯, x(N−1), x(N). The basic principle of the algorithm was as follows.

1. The delayed coordinate method of phase space was used to reconstruct the phase space of the original signal for the one-dimensional sequence *X*(*i*), and continuous m points were extracted from each sample point. Then, the m-dimensional space reconstruction vector of *X*(*i*) was obtained according to the following Formula (5), where m is the embedding dimension and *t* is the delayed time:

(5) $$X_{i}=[x(i),x(i+t),\cdots ,x(i+(m-1)*t)]$$

2. The phase-space matrix of the signal sequence was as follows:

(6) $$x=\left\{\begin{array}{l} X1\\ \vdots \\ X_{n-ml+l} \end{array}\right\}$$

3. The elements in the reconstructed vector of *x*(*i*) were arranged in ascending order using Formula (7):

(7) $$x\left(i+\left(j_{1}-1\right)t\right)\leq x\left(i+\left(j_{2}-1\right)t\right)\cdots \leq x\left(i+\left(j_{m}-1\right)t\right)$$

where $j_{1},j_{2},\cdots \cdots ,j_{m}$ is the column index of each element of the reconstructed component. The sequences of symbols for any row of the matrix were obtained by reconstruction of *X*(*i*), as in Formula (8):

(8) $$S(l)=\left(j_{1},j_{2},L,j_{m}\right),l=1,2,\cdots ,k,\;k\leq m!$$

4. Finally, the occurrence frequency of all permutations in the sequence was calculated, with the permutation entropy of the normalized sequence defined as in Formula (9):

(9) $$H(m)=\left(-\sum_{i=1}^{k}p_{i}\cdot \mathrm{lg}p_{i}\right)\mathrm{lg}(m!)$$

SampEn was used to measure the order of a time series. When the value of SampEn is larger, the complexity of the corresponding EEG signal is higher. The basic principle of the algorithm is as follows [28].

the signal sequence including *N* points is assembled into a m-dimensional vector

$$X_{m}\left(i\right)=\left(x\left(i\right),x\left(i+1\right),\ldots ,x(i+m-1\right),1\leq \;i\leq N-m+1$$

1. The distance between two vectors is denoted as

d[X(i),X(j)]=max|x(i+k)−x(j+k)|

2. Setting the tolerance limit r(r>0) and defining 1≤i≤N−m, the number of $d\left[X_{m}\left(i\right),X_{m}\left(j\right)\right]<r$ is calculated, then the ratio of the result to N−m−1 is defined as follows:

$$B_{i}^{m}\left(r\right)=\frac{1}{N-m-1}num\left\{\;\left[d\left[X_{m}\left(i\right),X_{m}\left(j\right)\right]<r\right]\right\},1\leq i,j\leq N-m,i\neq j$$

3. The average value is calculated as follows:

$$B_{m}\left(r\right)=\frac{1}{N-m}\sum_{i=1}^{N-m}B_{i}^{m}\left(r\right)$$

4. Similarly, the vector of (*m* + 1) dimensions is obtained as in steps 1 to 3 above:

$$A_{m}\left(r\right)=\frac{1}{N-m}\sum_{i=1}^{N-m}A_{i}^{m}\left(r\right)$$

5. The sample entropy of this signal sequence is defined as

$$SampEn\left(m,r\right)=\underset{n\to \infty }{\lim}(-ln\frac{A_{m}(r)}{B_{m}(r)})$$

6. Finally, as the actual calculation takes a limited value, the above equation can be valued as

$$SampEn\left(m,r,N\right)=-\ln\frac{A_{m}\left(r\right)}{B_{m}\left(r\right)}$$

LZC indicates the occurrence rate of the new pattern in the time series of an EEG signal, and the value of LZC is proportional to the occurrence rate of the new pattern [29,30]. The time series of A (al, a2, a3, …, an,) is known, allowing the average b of A to be calculated. Then, each value of A can be compared with b; if it is greater than b, it is set to 1, otherwise it is set to 0. Then, a new 0–l string is reconstructed. Assuming that the given string (c1, c2, c3, …, cn) has been reconstructed according to the rule, if cr is newly inserted it is not simply obtained by copying (c1, c2, c3, …, cr−1), but rather by C = (c1, c2, c3, …, cr). The last added point indicates the insertion. We can ask whether D = cr + 1 is included in string C; if true, it can be obtained simply by copying the symbol from C. This is equivalent to the question of whether DD is included in CDπ, where CDπ denotes a string consisting of C and D and π denotes that the last digit must be deleted (i.e., CDπ = C). The mean values of the four 10 s time-windows of each of the features were calculated as the final values of the signals.

### 2.3. Statistical Analysis

Nonlinear characteristics, including PmEn, SampEn, and LZC, were analyzed using the paired-samples *t*-test, independent-samples *t*-test, and one-way analysis of variance in SPSS statistical software for Windows, version 19 (SPSS, Chicago, IL, USA). A value of *p* < 0.05 was considered statistically significant.

## 3. Results

### 3.1. Temporal Aspect Analysis

This study first compares differences between stimulation, pre-stimulus, post-stimulus, and pre-stimulation in the EEG nonlinear characteristics of Alzheimer’s patients with different degrees of dementia. This analysis is based on the comparison of different temporal states, and is therefore a temporal aspect analysis.

#### 3.1.1. Temporal Aspect Whole Brain Area Analysis

The average value of the 59-channel EEG (with 5 of 64 channels being EMG and ECG) represents the characteristics of the whole brain regions.

The ratio between PmEn during stimulus and PmEn pre-stimulus is defined as the RcP. RcP reflects the changes in nonlinear dynamic features pre-stimulus and during stimulus. RcP between mild-to-moderate and severe patients was performed using the independent-samples t-test. In terms of the temporal aspect of whole brain regions, RcP during music stimulus between mild-to-moderate patients and severe patients showed a statistical difference (*p* = 0.017). RcP post-stimulus also showed a statistical difference (*p* = 0.009) between mild-to-moderate patients and severe patients, as shown in Table 2. Therefore, in terms of the temporal aspect of whole brain regions, brain responses to music stimulation between mild-to-moderate patients and severe patients showed a significant difference.

**Figure 2:**
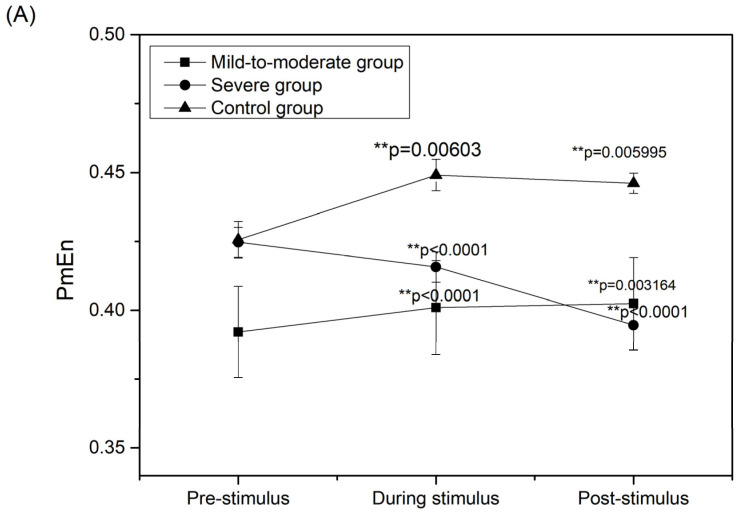

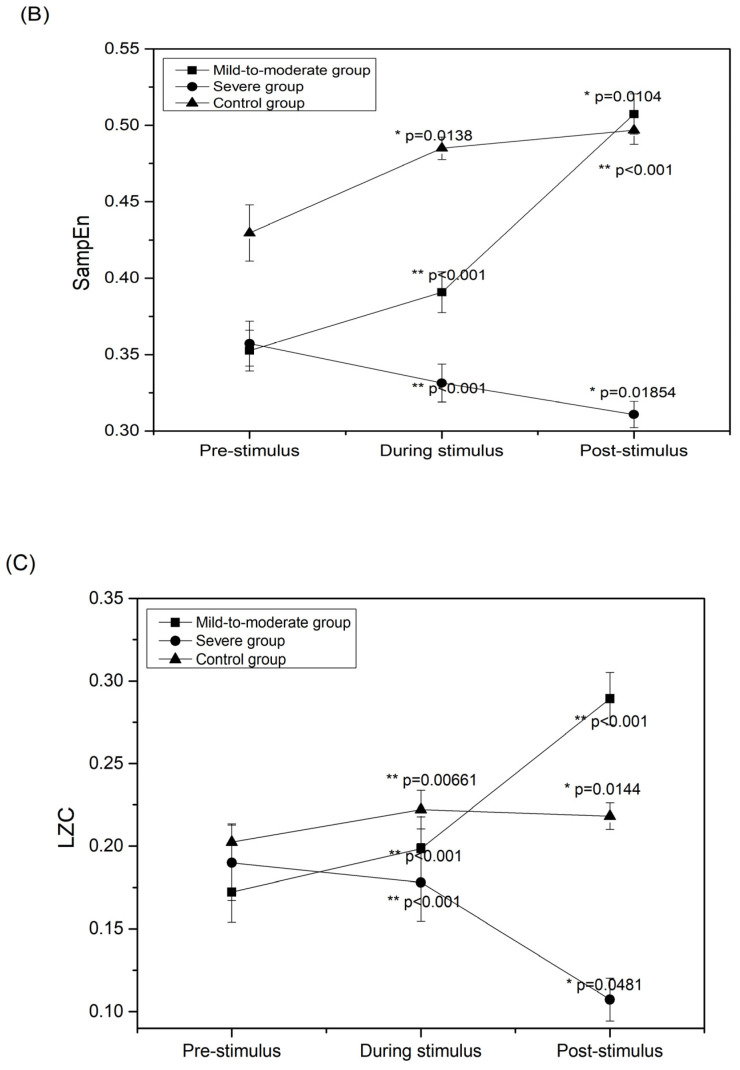
Results of temporal aspect variation of EEG nonlinear characteristic in whole brain region: (**A**) PmEn; (**B**) SampEn; (**C**) LZC. * *p* < 0.05, ** *p* < 0.01.

The specific results of PmEn at the temporal aspect for the whole brain area are as follows, and are shown in Figure 2A.

1. Mild-to-moderate patients showed higher entropy values during music stimulus compared to pre-stimulus (*p* < 0.0001) and higher entropy values at post-stimulus compared to pre-stimulus (*p* = 0.003164). Previous studies have shown that entropy values and complexity are both higher in people with normal cognitive abilities compared to those with Alzheimer’s disease. Therefore, the higher entropy during music stimulus compared to pre-stimulation in mild to moderate patients may reflect increased EEG activity and an improvement in cognitive level.
2. The entropy of severe patients showed lower values during stimulation compared to pre-stimulation (*p* < 0.0001), and lower entropy values post-stimulation compared to pre-stimulation (*p* < 0.0001). Related studies have shown that insomnia symptoms accompanied by anxiety have reduced EEG nonlinear characteristics when relieved [13], which may indicate a reduction in brain activity as well as relief from anxiety. Therefore, the decrease in entropy during music stimulation in severe patients compared to pre-stimulation may reflect the relief of anxiety.
3. The entropy values in the control group showed a similar trend as in the mild-to moderate group, with significantly higher entropy values during stimulus than pre-stimulation (*p*=0.00603) and significantly higher entropy values post-stimulation compared to pre-stimulation (*p* = 0.005995). Thus, cognitive-related brain responses may have been present in the control group under music stimulation as well.

The temporal level results for whole brain regions based on SampEn and LZC are similar to PmEn. The significance markers for the SampEn and LZC are shown in Figure 2B,C.

#### 3.1.2. Temporal Aspect Sub-Brain Area Analysis

In order to investigate whether the temporal aspect results of sub-brain regions (frontal, temporal, and parietal) during music stimulation are consistent with those of whole brain regions, this study investigates the temporal aspect results in sub-brain regions during music stimulation. One brain region, such as the frontal lobe, was fixed, and the EEG characteristics of the frontal lobe were compared with the differences between stimulation and pre-stimulation, post-stimulation, and pre-stimulation. The EEG characteristics of the frontal lobe were averaged over all channels headed by the letter “F”, allowing the temporal aspect results in the EEG characteristics of the parietal and temporal lobes to be obtained accordingly. As the occipital lobe is mainly responsible for visual processing, and this study involves music stimulation, which is related to auditory processing, in this paper the sub-brain results include three lobes (the frontal lobe, temporal lobe and parietal lobe).

**Figure 3:**
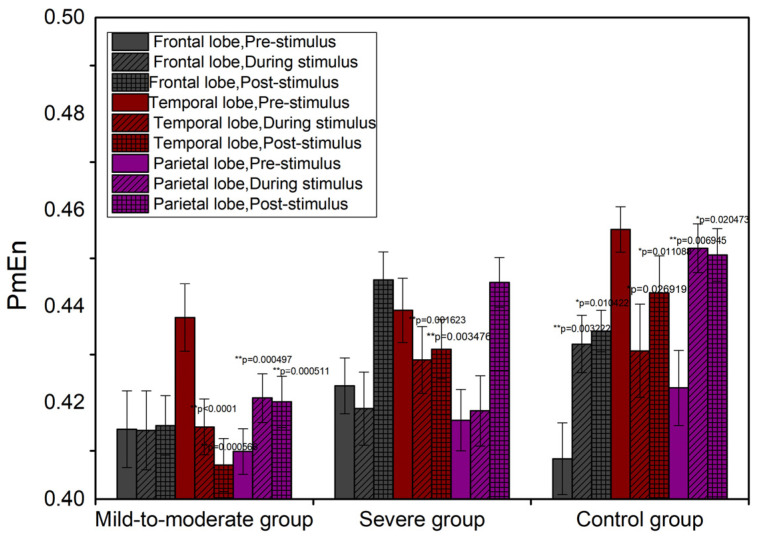
Temporal change of PmEn in sub-brain regions. * *p* < 0.05, ** *p* < 0.01.

Based on PmEn, independent samples t-tests were used to compare the number of brain lobes showing significant differences compared to pre-stimulus between mild-to-moderate and severe patients (*p* < 0.001). Thus, in terms of the temporal aspect of sub-brain regions, brain responses to music stimulation between mild-to-moderate patients and severe patients showed significant differences. The specific tendencies of PmEn compared to pre-stimulation in sub-brain regions are shown in Table 3, and the specific results are as shown in Figure 3. Compared to pre-stimulation, the regions where PmEn shows significant changes of values in mild-to-moderate patients were located in the parietal and temporal lobes (*p* < 0.01). There is a significant increase in entropy values in the parietal lobe during stimulation compared to pre-stimulation (*p* = 0.000497), and it is significantly higher post-stimulation compared to pre-stimulation (*p* = 0.000511). Previous studies have shown that the degree of cognition in Alzheimer’s disease is related to parietal lobe activity [31], and that entropy values and complexity gradually increase as cognitive abilities improve [26]. Therefore, the change in entropy over time in the parietal lobe can be interpreted as cognitive improvement in mild-to-moderate patients. Entropy values in the temporal lobe of mild to moderate AD decreased significantly (*p* < 0.0001) during stimulation compared to pre-stimulation, as well as post-stimulation compared to pre-stimulation (*p* = 0.000566). As emotional dysregulation is associated with abnormal brain activity in the right middle temporal gyrus [32], the temporal lobe may have the function of regulating emotions; therefore, this change in entropy in the temporal lobe can be interpreted as a relief of emotions in mild-to-moderate patients. The PmEn in the severe group showed significant differences during musical stimulation only in the temporal lobe, as shown by lower entropy values in temporal lobe stimulation compared to pre-stimulation (*p* = 0.001623) and lower entropy values post-stimulation compared to pre-stimulation (*p* = 0.003476), which may be interpreted as emotional regulation and relief of anxiety during music stimulus. Compared to pre-stimulation, PmEn in the frontal, temporal, and parietal lobes of the control group all differed significantly during musical stimulation, with the changes and significance labeling are shown in Figure 3. The only difference between the control group and the mild-to-moderate patients is that in addition to the parietal lobe and temporal lobe, PmEn in the frontal lobe of the control group changed significantly during music stimulation. This is because the frontal lobe is responsible for superior cognitive functions [33]; the control group under music stimulation shows superior cognitive responses in addition to simple cognitive responses and emotional responses.

**Figure 4:**
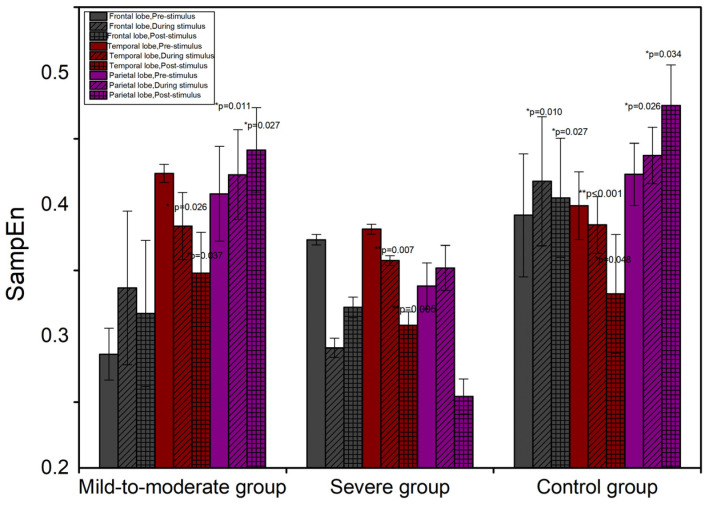
The temporal change of SampEn in sub-brain regions. * *p* < 0.05, ** *p* < 0.01.

**Figure 5:**
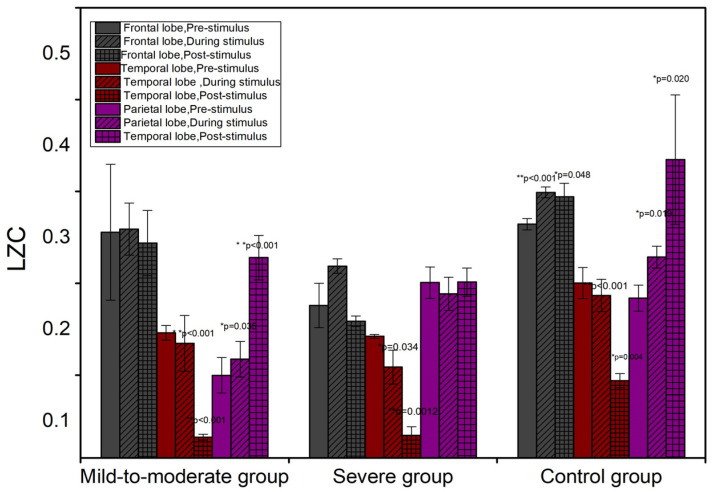
The temporal change of LZC in in sub-brain regions. * *p* < 0.05, ** *p* < 0.01.

The patterns of SampEn and LZ complexity are similar to that of PmEn. The tendencies of SampEn and LZC in the sub-brain regions compared to pre-stimulus are shown in Table 4 and Table 5. The temporal aspect changes of SampEn and LZC in the sub-brain regions are shown in Figure 4 and Figure 5.

**Figure 6:**
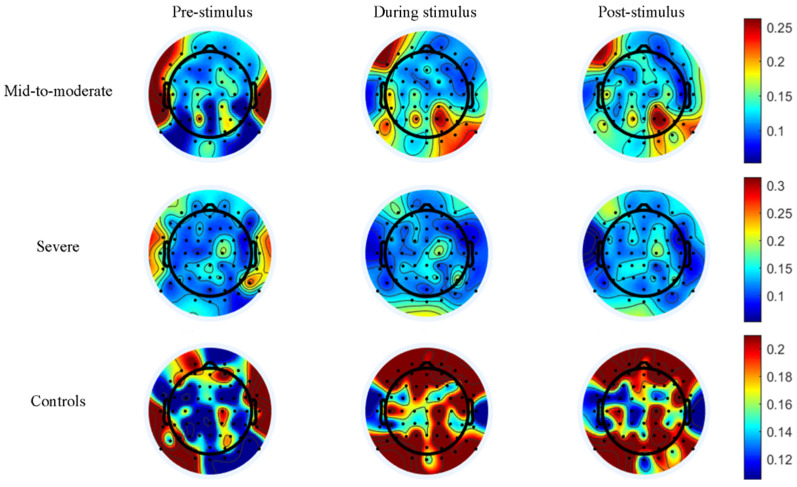
EEG topographic of the temporal changes in LZC in the sub-brain regions.

To visualize the temporal aspect of sub-brain regions’ response to music stimulation, the EEG topographic results of LZ complexity are shown in Figure 6.

The color in the parietal lobe of mild-to-moderate patients shows a greater range of red parts during and post-stimulation compared to pre-stimulation. In addition, the temporal lobe region changes its hue from red to blue, indicating a significant change in activity in both the parietal and temporal brain regions in the mild-to-moderate patients under music stimulation. In contrast, only the temporal lobe shows a lighter color in the severe patients during and post-stimulation compared to pre-stimulation, indicating that only the temporal lobe brain response is significantly altered in the severe patients. On the other hand, the color in the EEG topographic for the control group changes in all three brain regions, including the frontal, temporal, and parietal lobes, which indicates that the response of all three brain lobes is significantly altered in the control patients.

### 3.2. Spatial Aspect Analysis 

In terms of temporal aspect analysis, it was found that brain responses to musical stimulation show differences between mild-to-moderate patients and severe patients, suggesting that musical stimulation may have different effects on patients with different degrees of dementia. In order to further prove this conclusion, we additionally investigated the spatial aspect of the EEG characteristics of Alzheimer’s patients during musical stimulation. Spatial aspect analysis is based on fixing a certain time state and observing the differences in EEG characteristics in different brain regions during that time state. Data were statistically analyzed using one-way ANOVA, and differences were considered statistically significant when *p* < 0.05.

#### 3.2.1. Spatial Aspect Analysis Pre-Stimulus

The differences in nonlinear characteristics among the spatial brain regions (frontal, temporal, and parietal) pre-stimulus are shown in Table 6, Table 7 and Table 8. The EEG characteristics of the mild-to-moderate group, severe group, and healthy control group did not show significant differences among all brain regions.

#### 3.2.2. Spatial Aspect Analysis During-Stimulus

Based on PmEn, independent samples t-tests were used to compare the number of pairs. The results showed significant differences among brain regions between mild-to-moderate patients and severe patients (*p* < 0.001). Therefore, it was found that brain responses to musical stimulation between mild-to-moderate patients and severe patients show significant differences.

**Figure 7:**
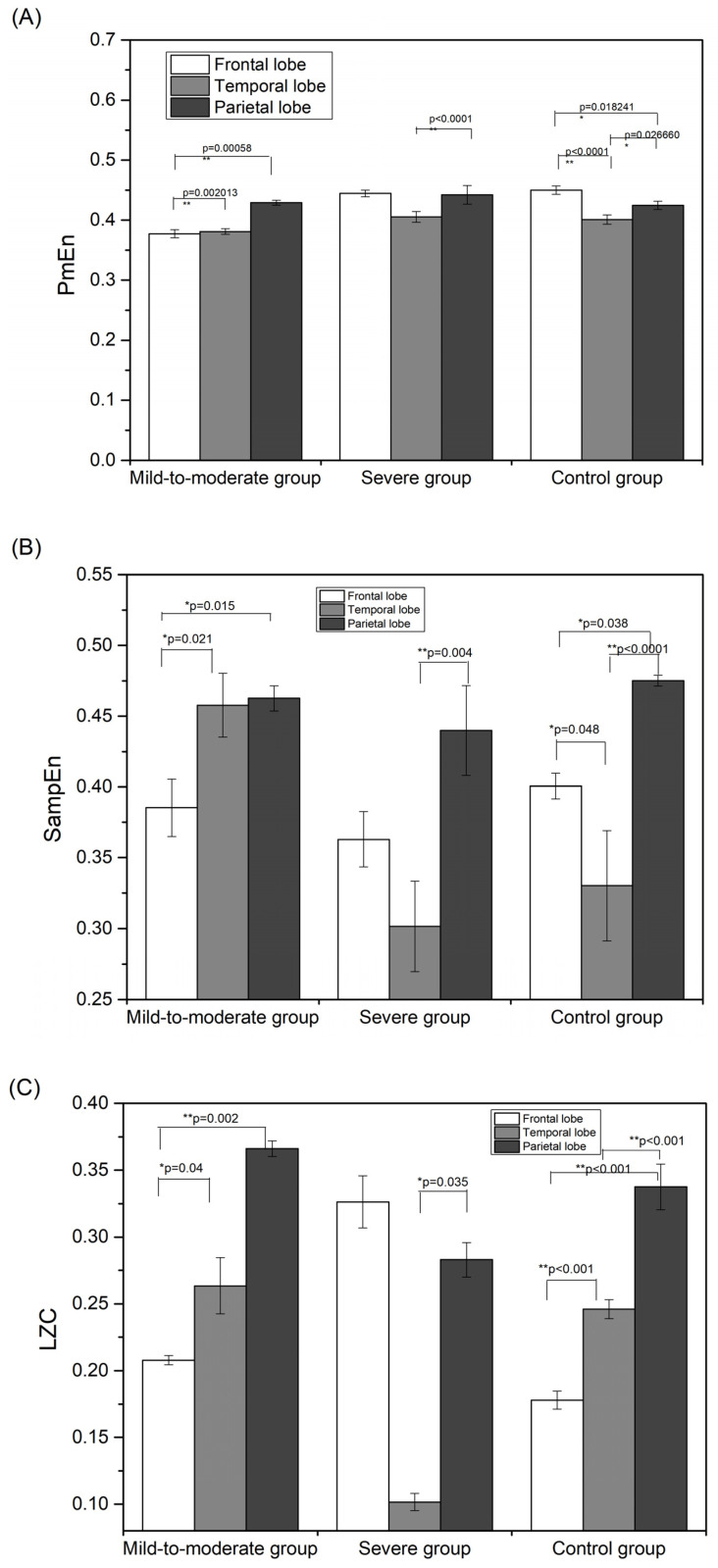
Spatial aspect results of nonlinear EEG characteristics during music stimulation: (**A**) PmEn; (**B**) SampEn; (**C**) LZC. * *p* < 0.05, ** *p* < 0.01.

The specific results of the spatial aspect of PmEn during stimulation are shown in Figure 7A.

(1) There were significant differences in PmEn among brain regions in the mild-to-moderate group (*p* = 000904), with a total of two significantly different pairs. The differences were demonstrated between the parietal and frontal lobes and between the temporal and frontal lobes. Entropy values were higher in the parietal lobe than in the frontal lobe (*p* = 0.00058), and significantly higher in the temporal lobe than in the frontal lobe (*p* = 0.002013). This correlates with the process of brain atrophy in Alzheimer’s disease [34,35]. For patients with mild to moderate dementia, the parietal lobe is not completely atrophied and the temporal lobe is functional; at this time, the neural signals of musical stimulation can be transmitted to the temporal lobe and the parietal lobe, and both the parietal and temporal lobes respond to the neural signals of musical stimulation. Thus, the results for PmEn show significant differences between the parietal and frontal lobe as well as between the temporal and frontal lobe in the different groups.

There were significant differences in PmEn among brain regions in the severe patient group (*p* < 0.0001), but only one set of significant difference pairs. The results only showed significantly lower entropy values in the temporal lobe compared to the parietal lobe (*p* < 0.0001). This is because for severe patients, the parietal and lateral temporal lobes are completely atrophied and only the medial temporal lobe remains functional; thus, the neural signals from music stimulation can only be transmitted to the temporal lobe, not to the parietal lobe, resulting the parietal lobe being unable to respond to the neural signals from music stimulation. As a result, the EEG PmEn of severe patients shows only one set of significant differences, namely, between the temporal lobe and the parietal lobe.

The PmEn in the control patients showed significant differences among brain regions (*p* < 0.0001), with three significantly different pairs. The values were significantly higher in the parietal lobe than in the frontal lobe (*p* = 0.026660), significantly lower in the temporal lobe than in the frontal lobe (*p* < 0.0001), and significantly higher in the frontal lobe than in the parietal lobe (*p* = 0.018241), which reflects normal functioning of all brain regions in the control group.

The patterns of SampEn and LZC during musical stimulus is similar to that of PmEn. The results for the spatial aspect of SampEn and LZC during stimulus are shown in Figure 7B,C.

#### 3.2.3. Spatial Aspect Analysis Post-Stimulus

**Figure 8:**
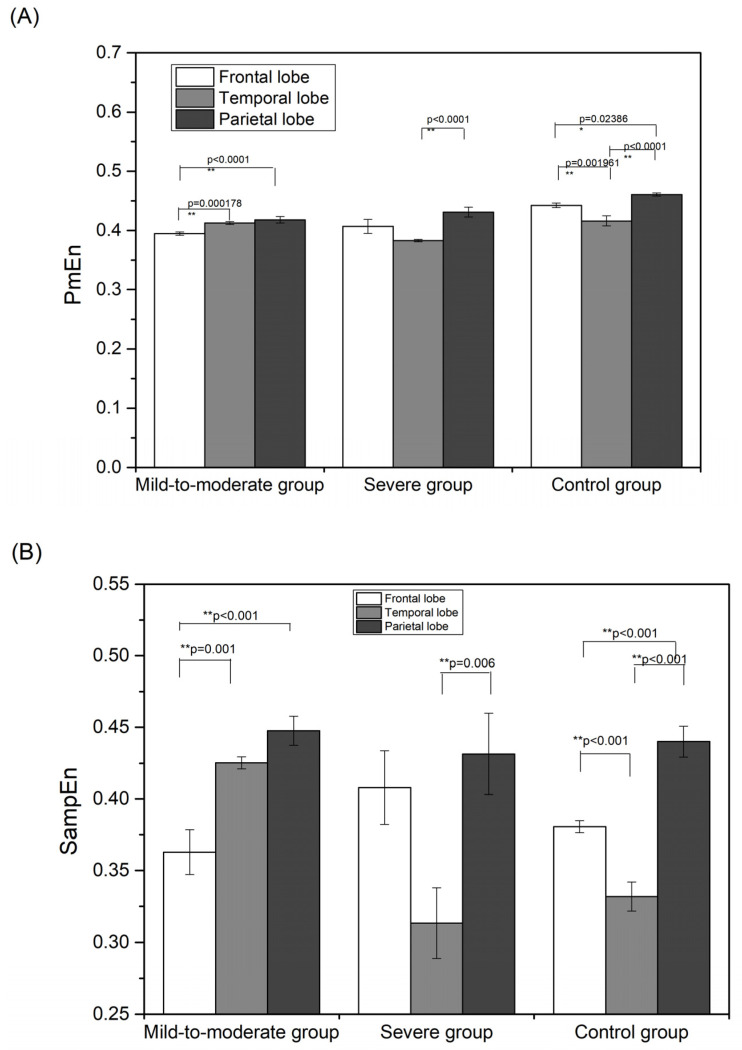

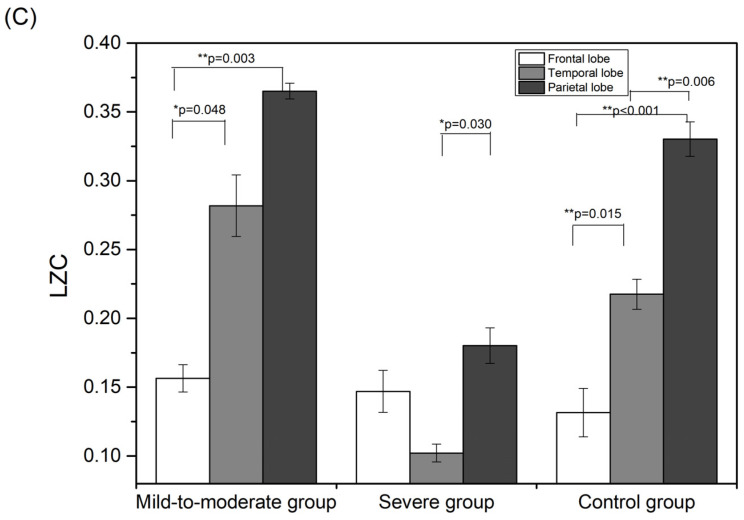
Spatial aspect results of nonlinear EEG characteristics post-stimulus: (**A**) PmEn; (**B**) SampEn; (**C**) LZC. * *p* < 0.05, ** *p* < 0.01.

The EEG non-linear characteristics of the spatial aspect post-stimulus are shown in Figure 8. It was found that brain responses to musical stimulation showed significant differences (*p* < 0.001) between mild-to-moderate patients and severe patients.

The PmEn, SampEn, and LZC in the mild-to-moderate patient group showed two significantly different pairs. The results showed significant differences between the parietal and frontal lobes as well as between the temporal and frontal lobes. Severe patients showed only one set of significant difference pairs, between the temporal and parietal lobes. The EEG nonlinear characteristics of the control group showed three sets of significantly different pairs.

## 4. Discussion

In this paper, significant differences in the spatial and temporal aspects were found in the EEG responses of Alzheimer’s disease patients with different degrees of dementia during music stimulation. The specific findings of this paper are as follows:

In the temporal aspect, the EEG responses of mild-to-moderate patients and severe patients showed significant differences at both the whole brain level and in sub-brain areas. It is significant that the brain areas with significant alterations during and post-stimulation compared to pre-stimulation are located in the parietal and temporal lobes in mild-to-moderate patients, while in severe patients the brain areas with significant alterations are located in the temporal lobes. These difference in the temporal aspect may indicate that music has different effects on patients with different degrees of dementia: both cognitive improvement and emotional relief were present in the mild-to-moderate patients, whereas only emotional relief and rarely cognitive improvement were present during musical stimulation in the severe patients. In the spatial aspect, the patients with mild-to-moderate and severe dementia showed different EEG responses, and fewer pairs of EEG characteristics showed significant differences between brain regions.

With respect to the above differences in spatio-temporal dynamics, few existing studies have provided an explanation for this difference. Our study attempts to account for this discrepancy using the theory of music cognitive hierarchical processing as well as the pathological progression of Alzheimer’s disease. According to the music cognitive processing model proposed by Koelsch et al., in 2011 [36], when physical sound is transmitted to the human ear, the first stage is the extraction of sound features. The basal nuclei and primary auditory cortex decode and analyze sound information to extract sound features such as frequency, timbre, intensity, and sound source. The second stage is the Gestalt formation stage. In this stage, sound features with similar frequency, rhythm, or timbre are reconstructed according to Gestalt rules, and the processing of melodic contour information is started. The third stage is interval analysis, which further analyzes the variations of melodic contour information based on the Gestalt fragments, as well as on the interval relationships between tones within chords or melodies, in order to characterize the intervals of melodies. Finally, the fourth stage is the processing of music structure analysis. In this stage, the brain further integrates the melodic information by analyzing the chord function and its relationships based on the principles of the tonal system.

According to the above music hierarchical processing theory and the results of our study, the perception of music in mild-to-moderate patients may be in the fourth stage of music processing, while the perception of music in severe patients may be in the third stage of music processing. This might be related to the pathological process of Alzheimer’s disease. The degree of brain atrophy in mild-to moderate patients differs from that in severe patients, resulting in differences in neural projections. The core area for stage 3 interval analysis may be located bilaterally in the superior temporal gyrus, whereas the core area for stage 4 structural analysis might be the prefrontal lobe, with a small activation in the inferior parietal lobe. Stages 3 and 4 transmit by way of the superior temporal gyrus, which is responsible for different stages of musical processing [37], i.e., there may be a neural projection from the superior temporal gyrus to the prefrontal lobe. Previous studies have shown that the process of brain atrophy in Alzheimer’s disease manifests as atrophy of the parietal and lateral temporal lobes followed by atrophy of the medial temporal lobe [34,35]. In mild-to-moderate patients, because the parietal lobe atrophies earlier and the temporal lobe functions relatively well, music signals can reach the fourth stage of music processing through the superior temporal gyrus, while in severe patients, temporal lobe atrophy is more severe and the music signals might not reach the fourth stage through the superior temporal gyrus. Therefore, the perception of music by severe patients may be in the third stage.

The significance of our findings is as follows. On the one hand, these findings have adjunctive implications for music therapy in clinical Alzheimer’s disease, allowing for more targeted treatment. For example, different approaches to music therapy could be utilized for patients with different degrees of dementia. On the other hand, the brain variations in Alzheimer’s patients in response to music stimulation might represent a model for investigating the neural mechanism of music perception.

However, our study has several limitations. The stage of music processing (interval analysis or structural analysis) at which mild-to-moderate and severe patients are situated requires validation in a broader study. In addition, fMRI, which has higher spatial resolution compared to EEG, should be performed to explore the activation areas of brain regions under music stimulation in order to validate the music processing hierarchy in AD patients with different degrees of dementia, which could lead to future theoretical research on the neural mechanisms of music perception.

## 5. Conclusions

In this paper, EEG data at pre-, during, and post-stimulation were selected in order to investigate the brain response to music stimulation of AD patients with different degrees of dementia. It was found that during music stimulation there are significant differences in PmEn, SampEn, and LZC in terms of their spatio-temporal dynamics between mild-to-moderate and severe patients. Our findings have adjunctive implications for music therapy in clinical Alzheimer’s disease, allowing for more targeted treatment. In addition, the brain variations of Alzheimer’s patients in response to music stimulation might represent a model for further investigation of the neural mechanisms of music perception.

## Figures and Tables

**Table 1:** Patient Information.

Group	Number	MMSE	BPSD	Average Age	Sex	
					Male	Female
Mild-to-moderate	17	17.5 ± 4.5	15.9 ± 9.0	80.2 ± 5.4	8	9
Severe	16	6.3 ± 4.3	18.3 ± 7.8	82 ± 4.3	8	8
Control	16	24.7 ± 2.2		81 ± 2.8	8	8

**Table 2:** RCP between mild-to-moderate and severe patients.

During Stimulus		Post Stimulus	
Mild-to-Moderate	Severe	Mild-to-Moderate	Severe
1.0401 ± 0.01822	0.9376 ± 0.03054	1.1.099 ± 0.4139	0.9628 ± 0.00859
*p* = 0.0017		*p* = 0.0090	

**Table 3:** Trend of PmEn compared to pre-stimulus in sub-brain regions.

PmEn	Frontal Lobe		Temporal Lobe		Parietal Lobe	
	During Stimulus	Post- Stimulus	During Stimulus	Post- Stimulus	During Stimulus	Post- Stimulus
Mild-to-moderate			↓	↓	↑	↑
Severe			↓	↓		
Controls	↑	↑	↓	↓	↑	↑

Note: “↑” represent that the value is significantly higher than that at pre-stimulus. “↓” represent the value is signififantly lower than that at pre-stimulus.

**Table 4:** Trend of SampEn compared to pre-stimulus in sub-brain regions.

SampEn	Frontal Lobe		Temporal Lobe		Parietal Lobe	
	During Stimulus	Post- Stimulus	During Stimulus	Post- Stimulus	During Stimulus	Post- Stimulus
Mild-to-moderate			↓	↓	↑	↑
Severe			↓	↓		
Controls	↑	↑	↓	↓	↑	↑

Note: “↑” represent that the value is significantly higher than that at pre-stimulus. “↓” represent the value is signififantly lower than that at pre-stimulus.

**Table 5:** Trend of LZC compared to pre-stimulus in sub-brain regions.

LZC	Frontal Lobe		Temporal Lobe		Parietal Lobe	
	During Stimulus	Post- Stimulus	During Stimulus	Post Stimulus	During Stimulus	Post Stimulus
Mild-to-moderate			↓	↓	↑	↑
Severe			↓	↓		
Controls	↑	↑	↓	↓	↑	↑

Note: “↑” represent that the value is significantly higher than that at pre-stimulus. “↓” represent the value is signififantly lower than that at pre-stimulus.

**Table 6:** PmEn of each brain region in the pre-stimulation period.

PmEn	Frontal Lobe	Temporal Lobe	Parietal Lobe
Mild-to-moderate	0.4249 ± 0.00593	0.4460 ± 0.00933	0.4212 ± 0.00657
Severe	0.4142 ± 0.00705	0.4183 ± 0.01419	0.4029 ± 0.00591
Controls	0.4403 ± 0.00435	0.4469 ± 0.00600	0.4335 ± 0.00553

**Table 7:** SampEn of each brain region in the pre-stimulation period.

SampEn	Frontal Lobe	Temporal Lobe	Parietal Lobe
Mild-to-moderate	0.4083 ± 0.00423	0.4122 ± 0.00269	0.4132 ± 0.00476
Severe	0.3904 ± 0.00724	0.4036 ± 0.00276	0.4025 ± 0.00356
Controls	0.4257 ± 0.00327	0.4265 ± 0.00400	0.4222 ± 0.00332

**Table 8:** LZC of each brain region in the pre-stimulation period.

LZC	Frontal Lobe	Temporal Lobe	Parietal Lobe
Mild-to-moderate	0.2949 ± 0.00256	0.3132 ± 0.00369	0.3035 ± 0.00278
Severe	0.2676 ± 0.00502	0.2763 ± 0.02311	0.2656 ± 0.00271
Controls	0.3403 ± 0.00267	0.3459 ± 0.00340	0.3435 ± 0.00412

## Data Availability

The datasets analyzed during the current study are not publicly available due to patient privacy of, but are available from the corresponding author on reasonable request.

## References

[1] Wimo A., Guerchet M., Ali G.C., Wu Y.T., Prina A.M., Winblad B., Jönsson L., Liu Z., Prince M. (2017). The Worldwide Costs of Dementia 2015 and Comparisons with 2010. Alzheimers Dement..

[2] Nowrangi M.A., Lyketsos C.G., Rosenberg P.B. (2015). Principles and Management of Neuropsychiatric Symptoms in Alzheimer’s Dementia. Alzheimers Res. Ther..

[3] Canu E., Sarasso E., Filippi M., Agosta F. (2018). Effects of Pharmacological and Nonpharmacological Treatments On Brain Functional Magnetic Resonance Imaging in Alzheimer’s Disease and Mild Cognitive Impairment: A Critical Review. Alzheimers Res. Ther..

[4] Atiwannapat P., Thaipisuttikul P., Poopityastaporn P., Katekaew W. (2016). Active Versus Receptive Group Music Therapy for Major Depressive Disorder—A Pilot Study. Complement. Ther. Med..

[5] Lyketsos C.G., Olin J. (2002). Depression in Alzheimer’s Disease: Overview and Treatment. Biol. Psychiat..

[6] Gold C., Solli H.P., Krüger V., Lie S.A. (2009). Dose–Response Relationship in Music Therapy for People with Serious Mental Disorders: Systematic Review and Meta-Analysis. Clin. Psychol. Rev..

[7] Bruer R.A., Spitznagel E., Cloninger C.R. (2007). The Temporal Limits of Cognitive Change From Music Therapy in Elderly Persons with Dementia Or Dementia-Like Cognitive Nmpairment: A Randomized Controlled Trial. J. Music Ther..

[8] Clément S., Tonini A., Khatir F., Schiaratura L., Samson S. (2012). Short and Longer Term Effects of Musical Intervention in Severe Alzheimer’s Disease. Music Percept. Interdiscip. J..

[9] Byrns A., Abdessalem H.B., Cuesta M., Bruneau M., Belleville S., Frasson C. (2020). Eeg Analysis of the Contribution of Music Therapy and Virtual Reality to the Improvement of Cognition in Alzheimer’s Disease. J. Biomed. Sci. Eng..

[10] Johnson J., Cotman C., Tasaki C., Shaw G. (1998). Enhancement of Spatial-Temporal Reasoning After a Mozart Listening Condition in Alzheimer’s Disease: A Case Study. Neurol. Res..

[11] Li C., Liu C., Yang Y., Chou M., Chen C., Lai C. (2015). Adjunct Effect of Music Therapy On Cognition in Alzheimer’s Disease in Taiwan: A Pilot Study. Neuropsychiatr. Dis. Treat..

[12] Svansdottir H.B., Snædal J. (2006). Music Therapy in Moderate and Severe Dementia of Alzheimer’s Type: A Case—Control Study. Int. Psychogeriatr..

[13] Wu L., Wang X., Yang Y., Dong T., Lei L., Cheng Q., Li S. (2020). Spatio-Temporal Dynamics of Eeg Features During Sleep in Major Depressive Disorder After Treatment with Escitalopram: A Pilot Study. BMC Psychiatry.

[14] Kang J., Chung Y.G., Kim S. (2015). An Efficient Detection of Epileptic Seizure by Differentiation and Spectral Analysis of Electroencephalograms. Comput. Biol. Med..

[15] Stam C.J. (2005). Nonlinear Dynamical Analysis of Eeg and Meg: Review of an Emerging Field. Clin. Neurophysiol..

[16] Theiler J., Eubank S., Longtin A., Galdrikian B., Farmer J.D. (1992). Testing for Nonlinearity in Time Series: The Method of Surrogate Data. Phys. D Nonlinear Phenom..

[17] Jack C.R., Albert M., Knopman D.S., McKhann G.M., Sperling R.A., Carillo M., Thies W., Phelps C.H. (2011). Introduction to Revised Criteria for the Diagnosis of Alzheimer’s Disease: National Institute On Aging and the Alzheimer Association Workgroups. Alzheimers Dement. J. Alzheimers Assoc..

[18] Delorme A., Makeig S. (2004). Eeglab: An Open Source Toolbox for Analysis of Single-Trial Eeg Dynamics Including Independent Component Analysis. J. Neurosci. Meth..

[19] Flexer A., Bauer H., Pripfl J., Dorffner G. (2005). Using Ica for Removal of Ocular Artifacts in Eeg Recorded From Blind Subjects. Neural Netw..

[20] Comon P., Jutten C. (2010). Handbook of Blind Source Separation: Independent Component Analysis and Applications.

[21] Vigário R., Sarela J., Jousmiki V., Hamalainen M., Oja E. (2000). Independent Component Approach to the Analysis of Eeg and Meg Recordings. IEEE Trans. Bio. Med. Eng..

[22] Huang C., Wahlund L., Dierks T., Julin P., Winblad B., Jelic V. (2000). Discrimination of Alzheimer’s Disease and Mild Cognitive Impairment by Equivalent Eeg Sources: A Cross-Sectional and Longitudinal Study. Clin. Neurophysiol..

[23] Shayegh F., Sadri S., Amirfattahi R., Ansari-Asl K. (2014). A Model-Based Method for Computation of Correlation Dimension, Lyapunov Exponents and Synchronization from Depth-Eeg Signals. Comput. Meth. Prog. Biomed..

[24] Abásolo D., Hornero R., Gómez C., García M., López M. (2006). Analysis of Eeg Background Activity in Alzheimer’s Disease Patients with Lempel–Ziv Complexity and Central Tendency Measure. Med. Eng. Phys..

[25] Zhou Y., Huang R., Chen Z., Chang X., Chen J., Xie L. (2012). Application of Approximate Entropy On Dynamic Characteristics of Epileptic Absence Seizure. Neural Regen. Res..

[26] Li J., Shen J., Liu S., Chauvel M., Yang W., Mei J., Lei L., Wu L., Gao J., Yang Y. (2018). Responses of Patients with Disorders of Consciousness to Habit Stimulation: A Quantitative Eeg Study. Neurosci. Bull..

[27] Amigó J.M., Kennel M.B. (2007). Topological Permutation Entropy. Phys. D Nonlinear Phenom..

[28] Buzsaki G., Draguhn A. (2004). Neuronal Oscillations in Cortical Networks. Science.

[29] Cheng Q., Yang W., Liu K., Zhao W., Wu L., Lei L., Dong T., Hou N., Yang F., Qu Y. (2019). Increased Sample Entropy in Eegs During the Functional Rehabilitation of an Injured Brain. Entropy.

[30] Li C., Wang J. (2008). Similarity Analysis of Dna Sequences Based On the Generalized Lz Complexity of (0, 1)-Sequences. J. Math. Chem..

[31] Yang L., Yan Y., Li Y., Hu X., Lu J., Chan P., Yan T., Han Y. (2019). Frequency-Dependent Changes in Fractional Amplitude of Low-Frequency Oscillations in Alzheimer’s Disease: A Resting-State Fmri Study. Brain Imaging Behav..

[32] Guo Z., Liu X., Li J., Wei F., Hou H., Chen X., Li X., Chen W. (2017). Fractional Amplitude of Low-Frequency Fluctuations is Disrupted in Alzheimer’s Disease with Depression. Clin. Neurophysiol..

[33] Koch C. (2004). The Quest for Consciousness a Neurobiological Approach.

[34] Sluimer J.D., Karas G.B., Vrenken H., van Schijndel R.A., van der Flier W.M., Goekoop R., Rombouts S.A., Scheltens P., Barkhof F. (2006). Ic–P–053: Relevance of Temporo—Parietal Atrophy in Mci Conversion to Alzheimer’s Disease: A Voxel—Based Morphometry Study. Alzheimers Dement..

[35] Cho S., Minn Y., Kwon K. (2010). Lateral Temporal Atrophy in Mild Cognitive Impairment. Alzheimers Dement..

[36] Koelsch S. (2011). Toward a Neural Basis of Music Perception—A Review and Updated Model. Front. Psychol..

[37] Lai H., Xu M., Sony Y., Liu J. (2013). The Neural Mechanism Underlying Music Perception: A Meta-Analysis of Fmri Studies. Acta Psychol. Sin..

